# Effect of thermal therapy and exercises on acute low back pain: a protocol for a randomized controlled trial

**DOI:** 10.1186/s12891-020-03829-7

**Published:** 2020-12-05

**Authors:** Claudia Côté-Picard, Jean Tittley, Catherine Mailloux, Kadija Perreault, Catherine Mercier, Clermont E. Dionne, Jean-Sébastien Roy, Hugo Massé-Alarie

**Affiliations:** 1 Centre interdisciplinaire de recherche en réadaptation et intégration sociale (Cirris), Université Laval, 525 Boulevard Wilfrid-Hamel, Québec, QC G1M 2S8 Canada; 2 Département de réadaptation, Université Laval, 2325, rue de l’Université, Québec, QC G1V 0A6 Canada; 3 Centre de Recherche du CHU de Québec - Université Laval, 1050, chemin Sainte-Foy, Quebec, QC G1S 4L8 Canada

**Keywords:** Acute low back pain, Thermal therapy, Exercises, Randomized controlled trials

## Abstract

**Background:**

Low back pain (LBP) is the first cause of years lived with disability worldwide. This is due to the development of chronic pain. Thus, it is necessary to identify the best therapeutic approaches in the acute phase of LBP to limit the transition to chronic pain. Superficial heat presents the highest level of evidence for short-term reduction in pain and disability in acute LBP. Physical activity is also recommended to avoid transition to chronic LBP, but there is a lack of evidence to determine its effect to reduce acute LBP. Also, the long-term effects of these interventions are unknown. This is a protocol for a randomized controlled trial (RCT) to determine the short and long-term effects of wearable continuous low-level thermal therapy, in combination with exercises or not, on disability and pain.

**Methods/design:**

Sixty-nine participants with acute LBP will be randomly assigned to one of three intervention programs: 1) thermal therapy, 2) thermal therapy + exercises, and 3) control. The interventions will be applied for 7 continuous days. The primary outcome will be disability and secondary outcomes will be pain intensity, pain-related fear, self-efficacy, number of steps walked and perception of change. The evaluators will be blinded to the interventions, and participants will be blinded to other groups’ interventions. Primary and secondary outcomes will be compared between intervention groups.

**Discussion:**

This study will provide new evidence about acute LBP treatments, to limit transition to chronicity. This will be the first study to measure the long-term effects of wearable continuous low-level thermal therapy, combined or not to exercises.

**Trial registration:**

This RCT has been retrospectively registered on ClinicalTrials.gov (NCT03986047) on June 14th, 2019.

## Background

Low back pain (LBP) produces a huge individual and socioeconomic burden through direct and indirect costs, such as patients’ management (imaging, drugs, etc.) and productivity loss, respectively [1, 2]. It is the first cause of years lived with disability in many countries [3]. A study reported that 12 months after LBP onset, pain was still present in 62% of patients [4]. This shows the necessity to find the best treatment approaches in the acute phase in order to prevent the transition from acute to chronic LBP (ALBP/CLBP). The latest clinical guidelines from the “American College of Physicians for ALBP [5]” recommends to select non-pharmacological treatments in the acute phase like superficial heat, massage, vertebral manipulations and acupuncture. The application of superficial heat (“thermal therapy”) presents the strongest evidence (moderate) for short-term reduction in pain [5–8]. Randomized controlled trials (RCT) [9–11] measured the effect of a wearable heatwrap (the most studied form of thermal therapy in ALBP). The results showed that it reduces pain and disability significantly up to 2 days after the end of the intervention in people with acute LBP [9–11]. This effect was superior to ibuprofen, acetaminophen or an oral placebo [11]. Although heatwrap reduces pain in the acute phase of LBP, its long-term effect is unknown. The benefit of this passive treatment may not transfer on the long-term. Active treatment such as exercises and avoiding bed rest could be better suited to influence pain and disability on the long-term. For example, avoiding bed rest is recommended to prevent CLBP [12] and exercises are consistently endorsed by guidelines and systematic reviews to treat CLBP [6, 13]. However, exercises alone have not been shown effective in ALBP [5]. An RCT [9] evaluated the effect of continuous low-level thermal therapy combined with McKenzie exercises during 5 days in LBP patients. The results showed that it reduced pain and disability significantly more than exercises alone, thermal therapy alone or a placebo up to 2 days after the end of the intervention. Thus, the use of heatwrap may provide sufficient pain relief to remain active and better perform exercises, perhaps by reducing fear of movement and increasing self-efficacy. The specific contribution of the present study will be to determine whether the short-term effects of combining thermal therapy and exercises in ALBP transfer to the long-term.

### Objectives and hypothesis

Our principal objective is to determine the effect of wearable continuous low-level thermal therapy combined or not with exercises on disability, pain intensity, pain-related fear, self-efficacy and perception of change in short-term (i) and long-term (ii) in adults presenting ALBP. Our secondary objective is to determine the effect of these interventions on the number of steps walked over the first week of treatment. We hypothesize that the combination of thermal therapy and exercises will have a larger positive effect for all outcomes (disability, pain intensity, pain-related fear, self-efficacy and perception of change) compared to thermal therapy alone and the control group in the short and long-terms. It will also be associated with a higher number of steps walked during the first week of treatment for the group receiving a combination of thermal therapy and exercises suggesting a higher level of physical activity.

## Methods and analysis

### Study setting

The study will take place at the research center (Cirris) in Quebec City, Quebec, Canada.

### Participants

Sixty-nine adults between 18 and 65 years of age presenting ALBP (onset of low back pain with or without leg pain for less than 6 weeks [14]) who have had limited activities or daily routine for more than 1 day will be recruited. The recruitment will be realized through the Quebec Back Pain Consortium’s database, targeted email listings (e.g. Université Laval’s community) and primary care settings by the evaluator. Participants will be included if they score ≥ 10/100 on the Oswestry Disability Index (ODI) questionnaire. This cut-off was chosen to be able to measure a minimal clinically important difference (MCID) of 9 points in ALBP patients [15]). Participants will be excluded if they suffered from LBP in the 3 months preceding the current episode or if they present any sign of specific spinal pathology, like fracture, tumor, spinal infection and obvious signs/symptoms of peripheral and/or central neural impairment (e.g. severe self-reported weakness in lower limb muscles, bilateral reduced sensitivity and/or pins and needles of lower limb skin). They will be excluded if they have a history of lumbar spine surgery, fibromyalgia, rheumatoid arthritis, skin lesion in the lumbar area, alteration of cutaneous sensations or temperature perception, are pregnant or trying to become pregnant, or have cognitive impairments. Also, they will be excluded if they have had a recent change in medication that may influence pain (antidepressant, psychotropic drugs, opioids, etc.), if they had a cortisone infiltration in the last 6 weeks or if they present pain related to pregnancy.

### Study design

A single-blind (evaluator) parallel group RCT will be undertaken. Participants will be randomized to one of three groups: (1) thermal therapy, (2) thermal therapy + exercises, or (3) control; for a 1-week intervention program. Five assessment time points have been established (A_1_ to A_5_ – see Table 1).

**Table 1:** Study design

		Study period					
	Enrolment	Baseline	1-week intervention	Post-intervention			
**Time point**	**A**_**0**_	**A**_**1**_	**I**	**A**_**2**_	**A**_**3**_	**A**_**4**_	**A**_**5**_
**ENROLMENT:**							
**Initial screening**	**●**						
**Eligibility assessment**	**●**						
**Informed consent**	**●**						
**Treatment allocation**		**●**					
**INTERVENTIONS:**							
**Thermal therapy**			**●**				
**Thermal therapy + exercises**			**●**				
**Control**			**●**				
**ASSESSMENTS:**							
**Baseline demographic information**		**●**					
**Physical functioning** (ODI)		**●**		**●**	**●**	**●**	**●**
**Pain** (NRS)		**●**		**●**	**●**	**●**	**●**
**Pain-related fear** (TSK-11)		**●**		**●**	**●**	**●**	**●**
**Self-efficacy** (CDSES −6)		**●**		**●**	**●**	**●**	**●**
**Perception of change** (GRoC)				**●**	**●**	**●**	**●**
**Number of steps walked per day**			**●** (daily)				

A_0_: first contact with participants; A_1_: baseline assessment; I: 7-day intervention; A_2_: assessment 1 week after baseline; A_3_: assessment 4 weeks after baseline; A_4_: assessment 12 weeks after baseline; A_5_: assessment 24 weeks after baseline; ODI: ODI-French Canadian Version; NRS: pain numerical rating scale; TSK: 11-item version of Tampa Scale of Kinesiophobia; CDSES − 6: Chronic Disease Self-Efficacy Scale - Short-Version and GRoC: global rating of change

#### Randomization / blinding

The evaluator will be blinded to the participant’s intervention allocation and participants will be blinded to the nature of the interventions in the other groups. Thus the risks related to the application of heat will be explained only to the participants allocated to thermal therapy groups. The patients will also be unaware of the study objectives and hypotheses. A randomization list will be generated using a computer random number generator by an independent research assistant (allocation concealed in sealed and opaque envelopes) to attribute an intervention program to each participant. Stratification will be done by sex (because men and women may respond differently to interventions in LBP [16]) and ODI score: 1) between 10 and 19 and 2) 20 and more to ensure similar level of disability for each group.

#### Timeline

1. First visit: baseline measurements and intervention

*Baseline:* At the baseline assessment, demographic characteristics will be collected before participants complete self-administered questionnaires on symptoms and functional limitations (ODI-French Canadian Version) [15, 17], pain-related fear (11-item version of Tampa Scale of Kinesiophobia – TSK-11) [18] and self-efficacy measured with the Chronic Disease Self-Efficacy Scale - Short-Version (CDSES-6) [19]. Pain intensity will be measured using an 11-point numerical rating scale (NRS) [20, 21] (average previous week [1-week]). These 4 questionnaires will serve as outcome measures (see “Outcome measures” section). The STarT Back Screening Tool [22] and the Canadian minimal dataset for low back pain [23] will be completed to characterise the participants recruited. After baseline assessment*,* participants will be assigned randomly to one of three intervention programs.

*Intervention:* To start, participants will meet a physical therapist (PT) to receive their assigned program (60-min meeting). For participants assigned to the thermal therapy groups (thermal therapy alone and thermal therapy + exercises), a heatwrap (ThermaCare®, Pfizer inc., New York, USA) will be applied onto the lower lumbar spine area, whereas in participants assigned to the control group, a sham non-heat lumbar belt will be applied. In addition, the physical therapist will teach an exercise program to the participants assigned to the “thermal care + exercises” group.

2 Intervention program

After the 1st visit, participants will take part in their assigned 7-day intervention program. Every day, participants will indicate in a logbook their average pain level during the day and the adherence to their program. A fitness watch will be given to each participant to record the number of steps walked per day during intervention week. They will be asked to avoid meeting a health care professional for their ALBP during the 7 day-intervention program. After the 7 days, they will be free to do so.

3 Second visit – short-term follow-up

A 2nd visit will be realized approximatively 7 days after the 1st visit, and ODI, TSK-11, CDSES-6, average 1-week pain intensity and a global rating of change (GRoC) [24] will be measured to determine the short-term effect of thermal therapy.

4 Long-term follow-ups

At 4, 12 and 24 weeks after baseline assessment, the average 1-week pain intensity, TSK-11, ODI, CDSES-6 and GRoC will be completed electronically by each participant. Participants will also report the use of other interventions after the completion of the 7-day intervention (e.g. medication, psychological support, health care professional, exercises training).

### Intervention programs

All participants will take part in their assigned 7-day program and will receive the same advices: staying active, avoiding bed rest, activity modification and reassurance (see Additional file 1.)

1. *Thermal therapy group:* Participants will be asked to wear a heatwrap over the lower lumbar spine that heats up to 40 °C within 30 min. This temperature is maintained for 8 h [25]. The heatwrap will be worn during the day for 8 consecutive hours on 7 consecutive days.
2. *Thermal therapy + exercises group:* In addition to thermal therapy, participants will be asked to perform 30 min of exercises at home for 5 days. Four different categories of exercises will be given: 1) functional activity exposure (sitting, sit-to-stand, lifting, bending, etc.); 2) exercises targeting trunk muscles (motor and postural control exercises); 3) mobility of the lumbar spine and 4) preferential direction if applicable. The exercises will be selected by a physical therapist (PT) according to a pre-determined list of 23 different exercises (see Additional file 2 [26] and Additional file 3) after assessing the participant’s clinical history and performing a brief physical assessment. Thus each participant will have to perform a program of 3 tailored exercises among the different categories. A fourth exercise can be prescribed if the participant responds favourably to “preferential direction” exercise (meaningful reduction in pain intensity during the exercise). Each exercise will be usually performed for 2 or 3 series of 10 repetitions for a maximum of 30 min per day. Considering that no specific categories of exercises have been shown superior for LBP [6], these exercises have been selected to reflect clinical reasoning and practice.
3. *Control group:* a room temperature heatwrap will be worn during the day for 8 h on 7 consecutive days to control for potential supportive and sensory influence of the heatwrap.

### Outcomes

#### Primary outcome

The primary outcome of this study is disability, and will be evaluated by the ODI French Canadian Version [17]. This self-administered questionnaire contains 10 questions to answer on a 6-point Likert scale (0 = no pain-related disability to 5 = maximal pain-related disability); one to assess pain intensity and nine to determine how pain affects everyday life activities in different domains, for a total of 50 points reported in percentage (0–100%). It presents excellent construct validity (RMDQ) [27] (Pearson coefficient (r) = 0.84) and excellent test-retest reliability (intraclass correlation coefficient (ICC) = 0.92) [17]. As previously mentioned, the MCID for ALBP is 9 points [15].

#### Secondary outcomes

*Pain intensity* will be assessed by asking the participant to rate his average pain over the last 7 days on an 11-point numerical rating scale (NRS): (0 to 10), ranging from “no pain” to “worst imaginable pain” [28]. The NRS is commonly used to measure the effect of interventions in LBP patients [21] and has a MCID of 2 points [28]. Its strong association with the visual analog scale (VAS) (Pearson’s correlation coefficient (r) = 0.93 [20]), supports its excellent construct validity.

*Pain-related fear* will be assessed with the 11-item version Tampa Scale of Kinesiophobia (TSK-11) questionnaire. It measures problematic beliefs and behaviours associated with pain, focusing on beliefs that pain is damaging and that painful movements should be avoided [29]. Each item is self-rated on a 4-point Likert scale (1 = strongly disagree, 4 = strongly agree). The total score ranges from 11 to 44 points, and higher scores represent greater fear of re-injury due to movement [18]. For LBP, TSK-11 has good internal consistency (Cronbach’s alpha = 0.79) and test-retest reliability (ICC = 0.81) and has a standard error of measurement (SEM) of 3 points [18]. The strong correlation with the 17-items Tampa Scale of Kinesiophobia (TSK), r = 0.93, supports its construct validity [18].

*Self-efficacy* will be measured using the Chronic Disease Self-Efficacy Scale - Short-Version (CDSES-6), a 6-item version of the CDSES-33 (a questionnaire with 33 items) that measures the self-efficacy to adopt self-management behaviors and to achieve predefined goals [19]. Each item is self-rated on a scale from 1 (“not at all confident”) to 10 (“totally confident”) and the total score represents the mean of the 6 items. The CDSES-6 has a good internal consistency (Cronbach’s alpha (α) = 0.86) and has an excellent construct validity, demonstrated by a strong correlation with the CDSES-33 (r = 0.91) [19]. It has been proven sensitive to change in people with LBP [19].

*Perception of change* in global LBP condition since the initial assessment will be measured using a single-item question, the Global Rating of Change (GRoC). GRoC is measured on a 15-point Likert scale, ranging from − 7 to + 7, whereas − 7 means “a very great deal worse”, 0 means “about the same” and + 7 means “a very great deal better” [24, 30]. Test-retest reliability for LBP patients is fair to good (ICC = 0.61) [30] and the MCID is 3 points [30, 31].

*The number of steps walked* will be measured with a fitness watch (Garmin Forerunner 15 version 2.70, Garmin International Inc., Olathe, KS, U.S.A.), as a proxy of the level of physical activity. A recent study [32] challenged a similar device, the Garmin Vivofit 2 (Garmin International Inc., Olathe, KS, U.S.A.) to measure the number of steps walked. The results showed that it obtained small mean absolute percentage error values (< 3) for measuring the number of steps walked when compared to a video [33], reflecting its ability to measure accurately the number of steps walked. The fitness watches will be provided by the research center (Cirris.)

### Sample size & statistical analysis

The estimation of the sample size was done with G*Power 3.1.7 considering an effect size = 0.80; α = 0.05, 1- β = 0.80 and lost at follow-up = 10%. The sample size was calculated for the primary outcome (ODI) with data from an unpublished study in our group (*n* = 50 with ALBP), and revealed that 23 participants are required per group. Thus, 69 participants with ALBP will be recruited.

Generalized linear mixed models (GLMM) will be performed for all outcomes. Our GLMM will incorporate terms for Group and Time (0, 1, 4, 12 and 24 weeks), and Group x Time interaction with random effect for participants. The Group x Time interactions will inform if there is a benefit to combine both treatments. If a violation in assumptions allowing the use of GLMM occurs (e.g. a change in the types of data distribution between the different time points), a nonparametric longitudinal data (nparLD) analysis will be performed as this procedure provides robust rank-based methods, even in case of an unknown distribution or atypical measurements and outliers [34]. Both GLMM and nparLD work well with missing data without any need to impute or reject participants. A one-way ANOVA or a non-parametric Kruskal-Wallis ANOVA will be used to compare the effect of intervention on the number of steps walked per day. The significance threshold will be set at *p* < 0.05.

### Ethics and dissemination

Ethics approval was obtained from the *Comité d’éthique de la recherche sectoriel en réadaptation et intégration sociale, CIUSSS de la Capitale Nationale* (#2019–1731 – CER CIUSSS-CN) in March 2019. Any modification to the protocol will be submitted to the ethic committee for approval and to clinicaltrials.org. Written informed consent will be obtained from all of the participants, after they read the information brochure describing the project and the potential risks and benefits and after discussing with the investigator if they have any questions. Every participant will be informed that he/she is free to leave the project at any moment. All numeric data collected will be safely stored in files protected with a password, and hard copies will be kept in locked file cabinets. Only study investigators will have access to the data at the completion of the trial. Results of the RCT will be published in a peer-reviewed journal and de-identified data will be available on a public repository at the time of publication. Results will also be communicated through scientific meetings. The International Committee of Medical Journal Editors criteria for authorship will be followed and no professional writer will be involved.

### Harms

Participants will be asked to write down any minor side effects or adverse events that occurred through the 1-week intervention in the logbook. They will also have a phone number and an e-mail address to reach the PT if they experience any major adverse events or if they have any questions during the week. The PT will then be able to direct the participant to the appropriate resource or give appropriate advices.

## Discussion

This will be the first RCT to evaluate the long-term effect of thermal therapy, combined or not with exercises, in the treatment of ALBP. To date, it is known that thermal therapy [9] contributes to reduce pain in ALBP in the short-term [5] and its use is recommended in the latest Clinical Guideline for LBP management [5]. However, the effect of these interventions in the long term remains unknown. This lack of knowledge is problematic, because thermal therapy may only produce a transient analgesia, having no influence on the long term trajectory of LBP. Importantly however, thermal therapy might potentiate other interventions that could be more effective on the long-term, such as exercises. A better understanding of the time-dependent effect of thermal therapy and exercises in ALBP will allow improving the management of LBP, and thus help reduce the huge socioeconomic burden of this condition [1, 2].

## Supplementary Information

**Additional file 1:** Advices given to the participants. This is a translated (originally in French) detailed list of the advices given to all of the participants enrolled in the trial.

**Additional file 2:** Consensus on Exercise Reporting Template (CERT). This is a tool used to describe exercises in clinical trials. It describes the general characteristics of the exercise program.

**Additional file 3:** Exercises. Table including photos and complete description of each of the 23 exercises for the thermal therapy + exercises intervention group.

## Data Availability

De-identified data will be made available on a public repository at the time of publication.

## Abbreviations

ALBP/ CLBP: Acute/chronic low back pain
CDSES-6: Chronic Disease Self-Efficacy Scale - Short-Version
GRoC: Global rating of change
ICC: Intraclass correlation coefficient
MCID: Minimal clinically important difference
NRS: Numerical rating scale
ODI: Oswestry Disability Index
PT: Physical therapist
RCT: Randomized controlled trial
RMDQ: Roland-Morris Disability Questionnaire
TSK-11: 11-item version of Tampa Scale of Kinesiophobia
