# Supplementary material for: Effect of thermal therapy and exercises on acute low back pain: a protocol for a randomized controlled trial
Source: BMC Musculoskelet Disord. 2020 Dec 5;21:814. doi: 10.1186/s12891-020-03829-7 (PMC7719244; doi:10.1186/s12891-020-03829-7)
Supplement: Supplementary file 1 — Additional file 1: Advices given to the participants. This is a translated (originally in French) detailed list of the advices given to all of the participants enrolled in the trial. [file 12891_2020_3829_MOESM1_ESM.docx]

| **1) Natural evolution in ALBP** | Most people suffering from ALBP will see their condition improve naturally. The condition generally improves a lot in the first month after the onset of pain. |
| --- | --- |
| **2) Rest** | Complete bedrest (stay in bed all day) should only be done if the pain and incapacities are very severe, and it should not last more than 2 days. It is preferable to remain active according to your pain tolerance. |
| **3) Activities** | It is important to continue to perform your daily life activities, to continue to walk and work according to your tolerance and pain. A light pain that is felt while performing daily activities is not a sign that you are aggravating your condition. Simply, be reasonable and careful. Split the tasks that are demanding for your back to avoid an increase in pain. Take breaks frequently. |
| **4) Positions** | To help pain management, try to change positions (sitting, lying down, standing) every 30 minutes (except during the night). Be careful while taking a sitting posture with rounded back.  In sitting, LBP may be relieved by using a lumbar spine support (e.g. a small cushion or a rolled towel positioned at the level of the lower lumbar spine area) and avoid putting the feet up (e.g. on a footstool).  Avoid long car rides. If needed, stop the car after 30 or 45 minutes and get out of the car to walk for a few minutes before coming back in the car. |
| **5) In bed** | Avoid sitting up from lying down directly. Instead, before you get up, roll into side lying and then use your arms to push you in sit up position. Also, you can use a pillow between your knees when you are side lying or under your knees if you are lying on your back, if it reduces your pain. Avoid sleeping on your stomach. |
| **6) Medication** | In LBP, acetaminophen, anti-inflammatories or muscle relaxants decrease slightly the pain on the short term. However, it is not clear if they help to fix the problem on the medium or long terms. The current scientific knowledge suggests limiting their use if not necessary. |
| **7) To monitor** | In the case that at least one of the following symptoms appears, please contact rapidly a doctor or a qualified health care professional:   - Severe pain that is increasing for no specific reason; - Urinary or fecal incontinence; - Urinary retention (important difficulty to urinate) or constipation without a known reason; - Erectile dysfunction; - Loss or decreased sensitivity between the legs, at the genital organs or the buttock area; - Numbness and tingling in both legs. |

**Additional file 1**

**Advices given to the participants**
