# Supplementary material for: Effect of thermal therapy and exercises on acute low back pain: a protocol for a randomized controlled trial
Source: BMC Musculoskelet Disord. 2020 Dec 5;21:814. doi: 10.1186/s12891-020-03829-7 (PMC7719244; doi:10.1186/s12891-020-03829-7)
Supplement: Supplementary file 2 — Additional file 2: Consensus on Exercise Reporting Template (CERT). This is a tool used to describe exercises in clinical trials. It describes the general characteristics of the exercise program. [file 12891_2020_3829_MOESM2_ESM.docx]

**Additional file 2**

**Consensus on Exercise Reporting Template (CERT)** [26]

| Item Category | Item no. | Abbreviated Item Description |
| --- | --- | --- |
| **WHAT** : materials | 1 | **Type of exercise equipment**   - Yoga mat/ chair / kitchen counter |
| **WHO**: provider | 2 | **Qualifications, teaching/ supervising expertise, and/or training of the exercise instructor**   - PT |
| **HOW** : delivery | 3 | **Whether exercises are performed individually or in a group**   - Individually |
|  | 4 | **Whether exercises are supervised or unsupervised**   - The exercise program is supervised when taught to the participant, during the meeting with the PT, and it is unsupervised when the program is performed at home |
|  | 5 | **Measurement and reporting of adherence to exercise**   - The participants will have to write down in a logbook the number of series and repetitions of each exercise they performed at home every day |
|  | 6 | **Details of motivation strategies**   - Education about the importance to remain active in ALBP |
|  | 7 | **Decision rules for progressing the exercise program**   - Each participant will have pictures of different ways to perform each exercise and will be advised to progress the difficulty of the exercise if it doesn’t increase pain and if it became too easy to perform. |
|  | 8 | **Each exercise is described so that it can be replicated (eg, illustrations, photographs)**   - Illustrations and instructions will be given to the participants for each prescribed exercise (see **Additional file 3**) |
|  | 9 | **Content of any home program component**   - 3 or 4 types of exercises will be taught to the participants: 1) functional activity, 2) trunk muscle activation, 3) mobility of the lumbar spine and 4) “preferential direction” exercise if applicable |
|  | 10 | **Nonexercise components**   - Education regarding the natural evolution of ALBP, the importance to remain active and the best postures to adopt (see **Additional file 1**) |
|  | 11 | **How adverse events that occur during exercise are**  **documented and managed**   - It will be documented in the logbook, and the participants will be asked to stop the exercise if pain increases moderately to severely and they will have a telephone number and an e-mail address to reach the PT if they experience any adverse effect during home exercises |
| **WHERE** : location | 12 | **Setting in which exercises are performed**   - Exercises will be first performed at the research center with the PT and at home for the following days |
| **WHEN, HOW MUCH** : dosage | 13 | **Detailed description of the exercises (eg, sets, repetitions, duration, intensity)**   - 2 to 3 sets of 10 repetitions for each exercise without fatigue or pain, twice a day, for a total of 30 minutes per day |
| **TAILORING** : what, how | 14 | **Whether exercises are generic (“one size fits all”) or**  **tailored to the individual**   - Tailored to the individual, based on an assessment of the condition by the PT |
|  | 15 | **Decision rule that determines the starting level for exercise**   - Based on the assessment of the condition by the PT (active range of motion without pain, pain intensity at rest, initial capacities regarding functional movements, pain relief during exercises) |
| **HOW WELL** : planned, actual | 16 | **Whether the exercise intervention is delivered and**  **performed as planned**   - No particular measure will take place to ensure that the exercises are performed as taught, but the number of sets and repetitions will be documented) |
