# Supplementary material for: Effect of thermal therapy and exercises on acute low back pain: a protocol for a randomized controlled trial
Source: BMC Musculoskelet Disord. 2020 Dec 5;21:814. doi: 10.1186/s12891-020-03829-7 (PMC7719244; doi:10.1186/s12891-020-03829-7)
Supplement: Supplementary file 3 — Additional file 3: Exercises. Table including photos and complete description of each of the 23 exercises for the thermal therapy + exercises intervention group. [file 12891_2020_3829_MOESM3_ESM.docx]

**Additional file 3**

**Exercises**

| **A- Functional activity** | |
| --- | --- |
| Exercise - description | Illustration |
| 1. **Squat :**  - Place your hands on your thighs, and your feet at hip width; - Bend the knees and bend the trunk forward, keeping a straight alignment of the spine. Keep your knees aligned with your feet. - Bend your knees as much as you can without pain, or until you reach a position at which you could sit on a chair, then come back up. - If needed, you can put your hands on the back of a chair for more support. | 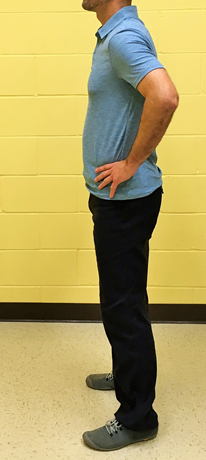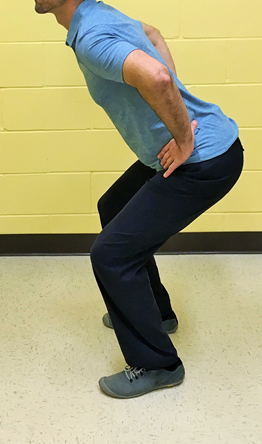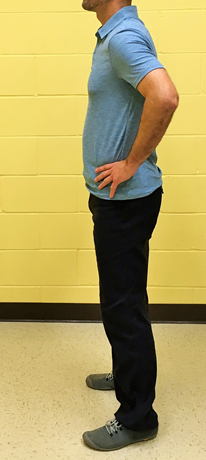 |
| 1. **Sit-to-stand**  - Start from a sitting position with your arms crossed on your chest. - Sit at the edge of the chair. - Bend the trunk forward, keeping a straight spine and push with your legs to stand up. - Once standing, bend your knees to go back slowly to a sitting position, with your arms still crossed on your chest. | **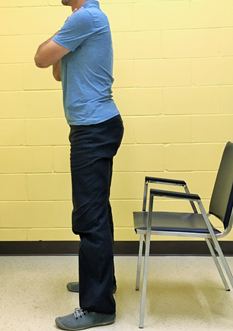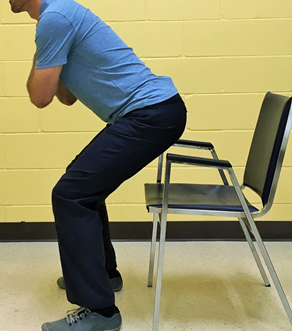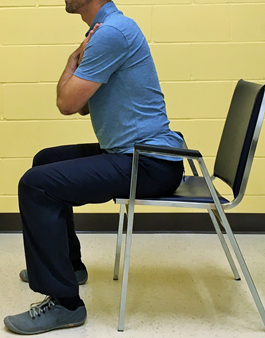** |
| 1. **Lifting**  - Lift a weight from the ground by bending the knees, with your feet at hip width, and by bending slightly your trunk forward, keeping a straight spine. - Come back up the same way. | **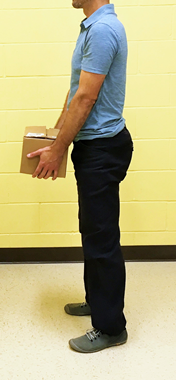**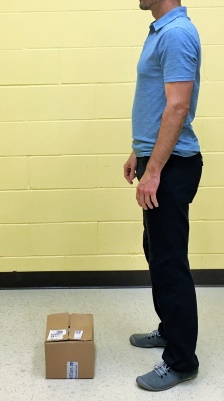**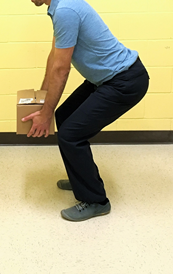**  **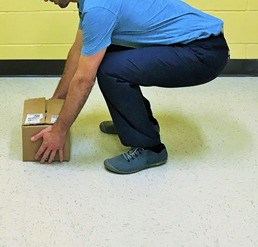** |
| **B- Trunk muscle activation** | |
| 1. **Abdominal muscles / transversus abdominis contraction**  - Lying on your back, with your knees bent, place your fingers approximately 2 cm inside of the pelvic bones. - Contract your pelvic floor and your transversus abdominis as if you wanted to stop urinating, or as if you wanted to bring your fingers closer to each other. During the contraction, continue to breathe normally. - Maintain the contraction for 5 to 10 seconds, relax, and start over again. | 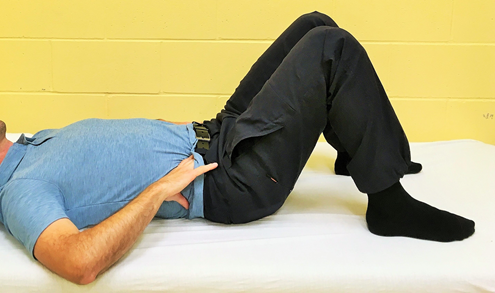 |
| 1. **Abdominal muscles / transversus abdominis** **contraction + leg movement**  - Contract your lower abdominal muscles as previously described. - Slide your heel on the mat to straighten your leg while maintaining an abdominal contraction, and come back to the initial position.   Or   - Lift your heel off of the mat while keeping your knee bent and your abdominal contraction, and come back slowly to the initial position.   Or   - Lift your heel off of the mat and straighten your leg without touching the mat, and come back slowly to the initial position in the same way. - For all the previous exercises, relax, contract your abdominals again and repeat with the opposite leg. | 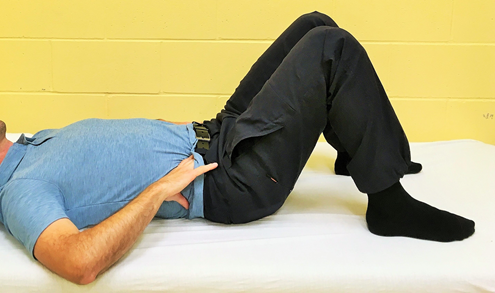  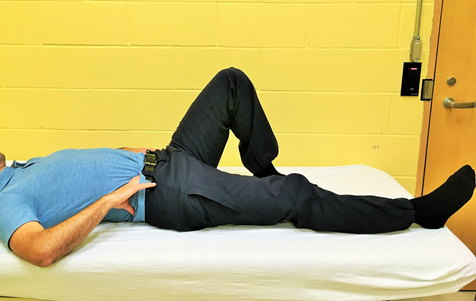    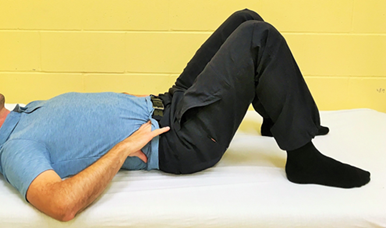Or  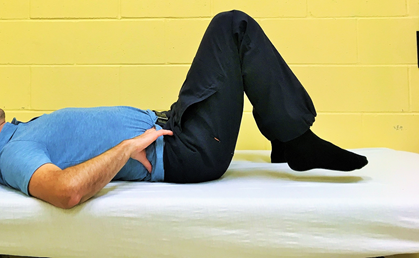    Or  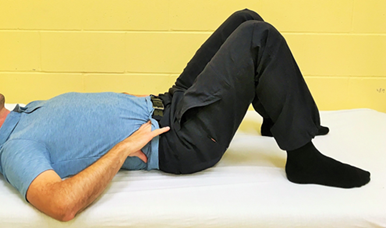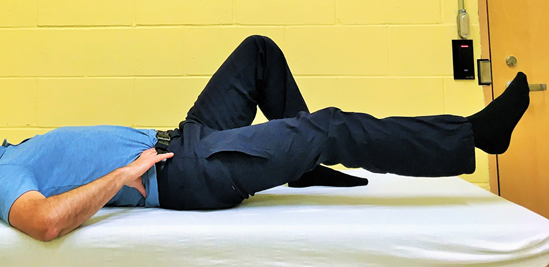 |
| 1. **Lumbar multifidus muscle activation**  - Lay down in a comfortable position – side lying or prone. - Place the tip of your fingers on the muscles located just outside the lumbar spine. - Contract softly to activate the muscles under your fingers and try to keep the other muscles relaxed. - Maintain the contraction for 5 to 10 seconds, relax, and start over again. | 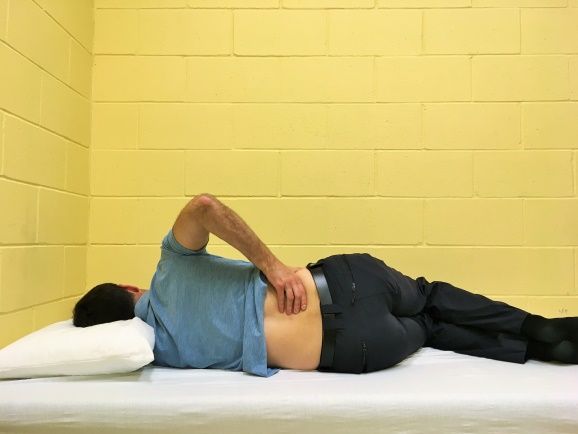 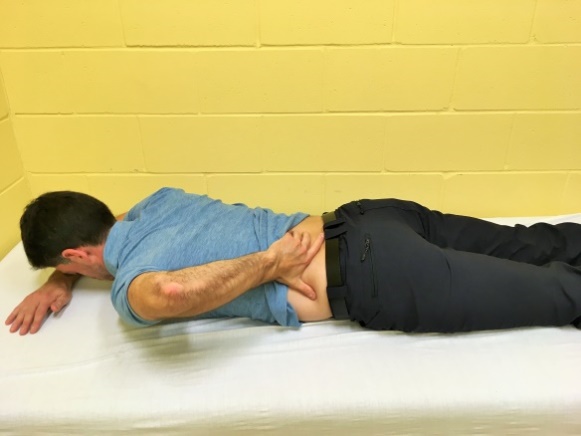 |
| 1. **Bird dog**  - Start in a 4-point kneeling position. - Lift one arm in front of you, parallel to the mat;   Or   - Lift one leg parallel to the mat;   Or   - Lift one arm and the opposite leg, both parallel to the mat; - Maintain for 2 seconds, and go back slowly to the initial position; - For all previous exercises, repeat with the opposite side. | 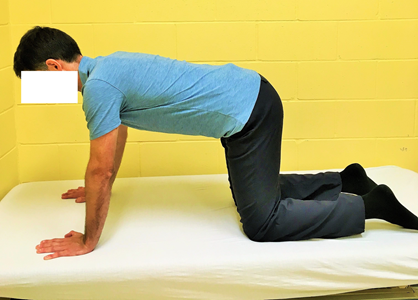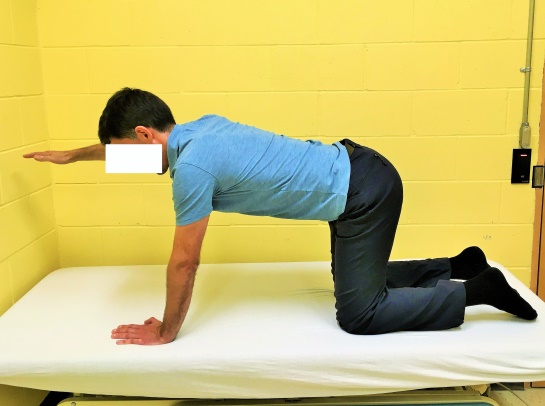  Or  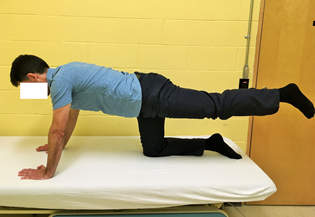  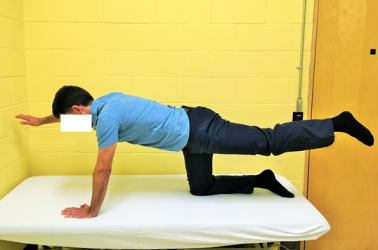Or  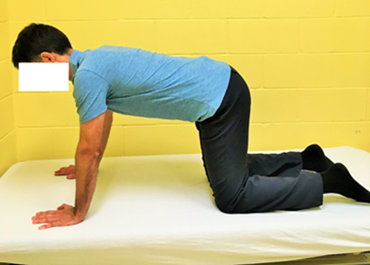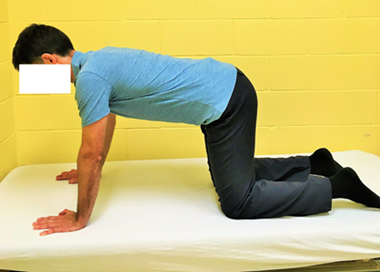 |
| 1. **Bridge**  - Start lying on your back with your knees bent. - Tighten your buttock and lift your pelvic up to reach a position where your trunk is aligned with your pelvic. - Maintain the position for 2 seconds and go back slowly to the initial position.   Or   - Once your pelvic is up and aligned with the spine, lift one leg and maintain for 2 seconds. Come back slowly to the initial position. - Relax, tighten your buttock again and start over (with the other leg if applicable.) | 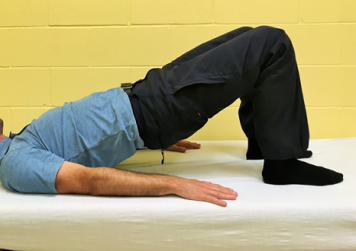  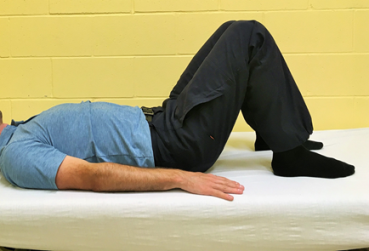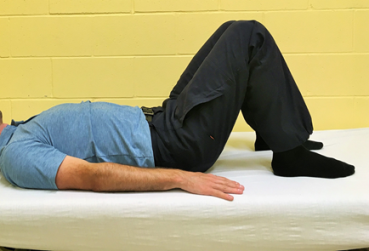Or  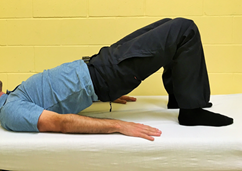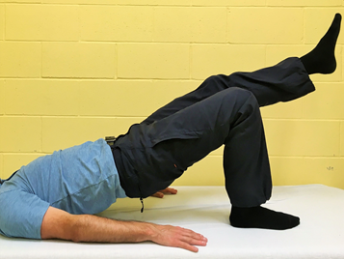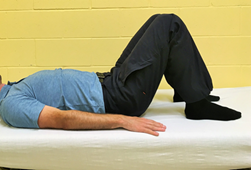 |
| 1. **Curl-up**  - Start lying on your back, with one knee bent and your hands placed in your low back. - Lift your head and upper back slightly while keeping your chin tucked and your head aligned with your trunk. - Maintain the position for 5 to 10 seconds. - Go back slowly to the initial position, relax and repeat. | 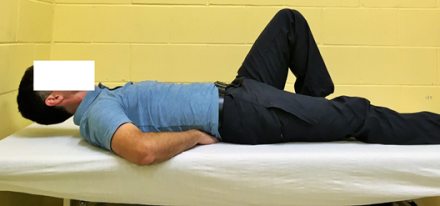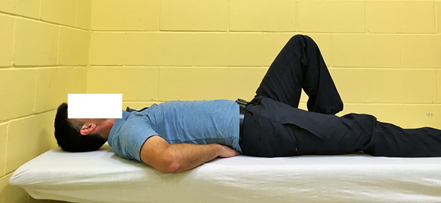 |
| 1. **Side plank**  - Start in a side lying position, with the elbow under the shoulder. - Lift your pelvic up so that your trunk and your pelvic are aligned. - You may do the exercise on your knees or on your feet. - Maintain for 15 to 30 seconds.   Or   - If these variations are too hard, you may do the exercise on a wall. Maintain for 15 to 30 seconds. | 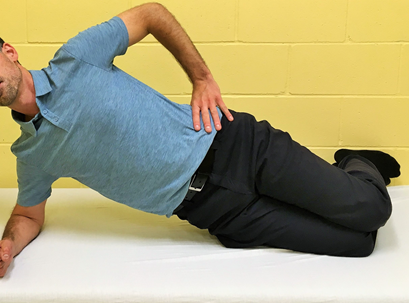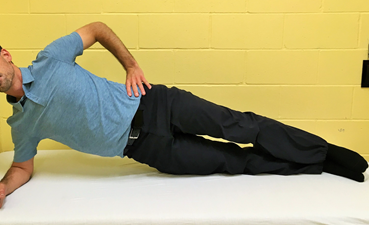  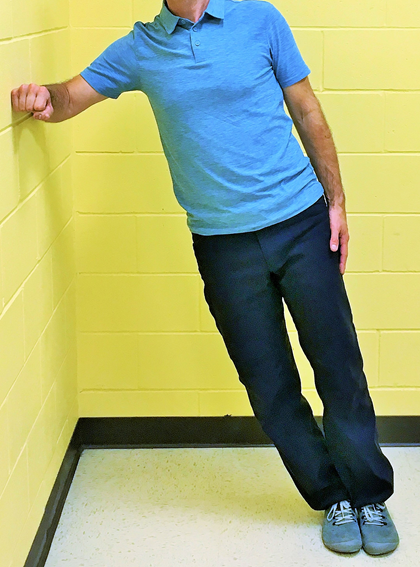 |
| 1. **Ventral plank**  - Start lying on your belly with your elbows under your shoulders. - Lift your pelvic up so that your body forms a line from your head to toes. - Variation: you may perform the exercise on your knees. - Maintain for 15 to 30 seconds.   Or   - Place your elbows on a chair that is against a wall and your knees on the ground. - Lift up your knees so that your body forms a line from your head to toes. - Variation: you may perform the exercise on your hands. - Maintain for 15 to 30 seconds. | 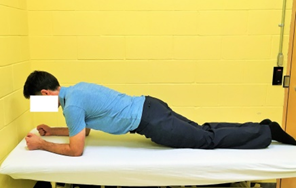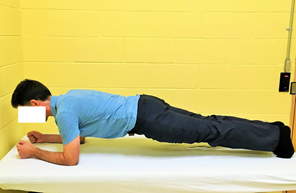  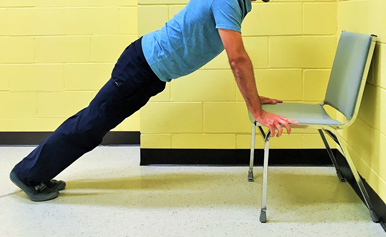Or  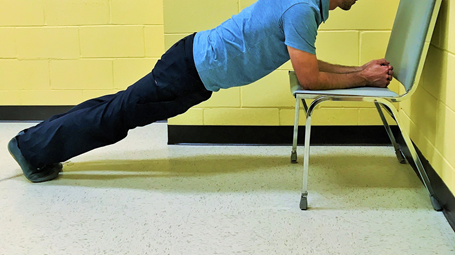 |
| 1. **Ventral plank (easier variations)**  - For easier variations of the plank, you may perform it with your elbows or hands on a counter or a wall instead of a chair. - Maintain for 15 to 30 seconds. | 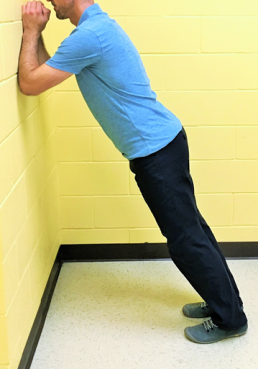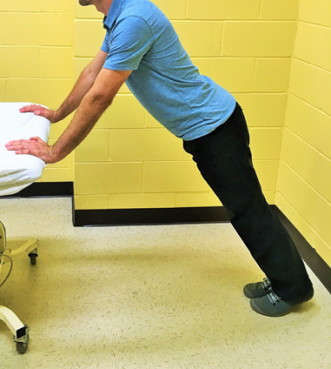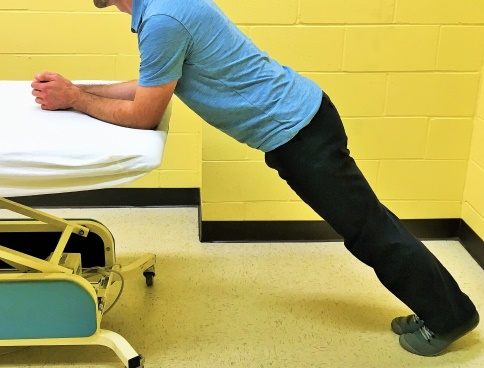 |
| 1. **Sitting and lifting a knee**  - Sit on a chair, your thighs parallel to the floor. - Lift slightly one knee without moving the pelvic or the lumbar spine. - Maintain the position for 5 to 10 seconds. - Put down your foot on the floor, and start over again with the other side. | 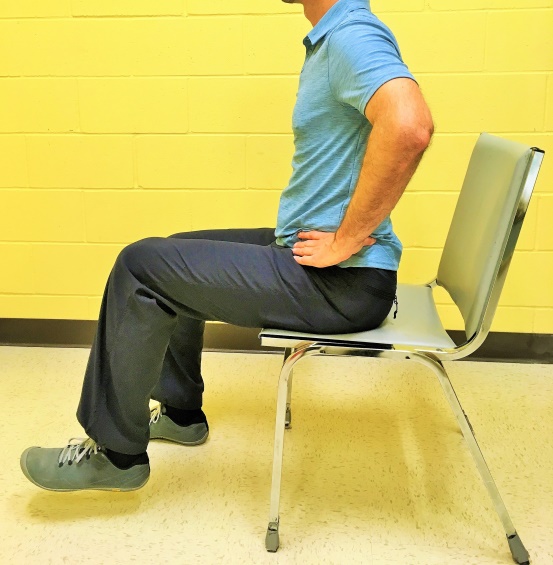  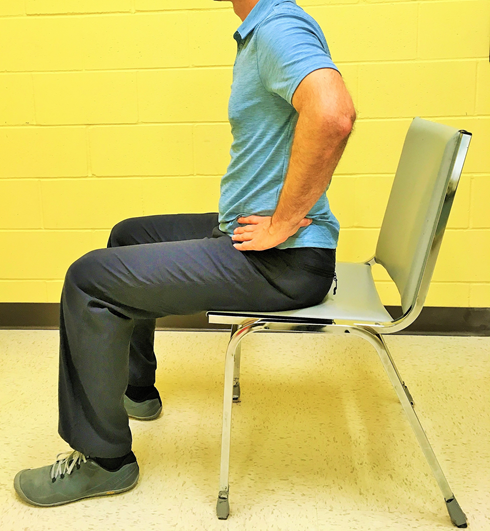 |
| **C- Mobility of the lumbar spine** | |
| 1. **Cat-Cow pose**  - Start in a 4-point kneeling position. - Round your spine up to the ceiling, with your chin tucked and then arch your back, alternatively. | **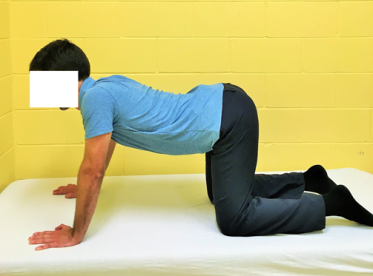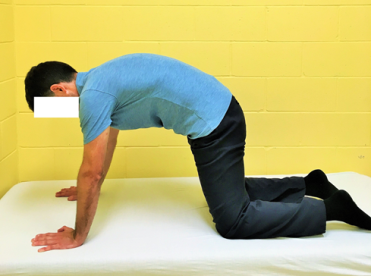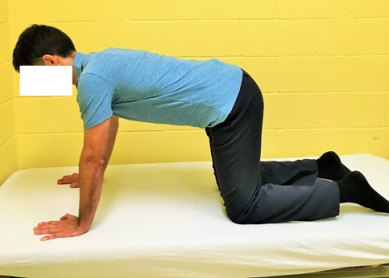** |
| 1. **Hip flexion supine**  - Start lying on your back, with your knees bent. - Bring one knee towards your belly and pull with your hands on your knee to feel a gentle stretch in your low back.   Or   - Bring both of your knees on your chest and pull with your hands on your knees to feel a gentle stretch in your low back. | 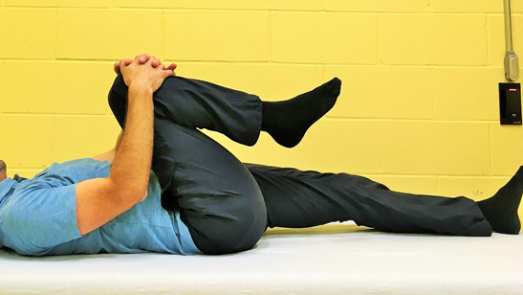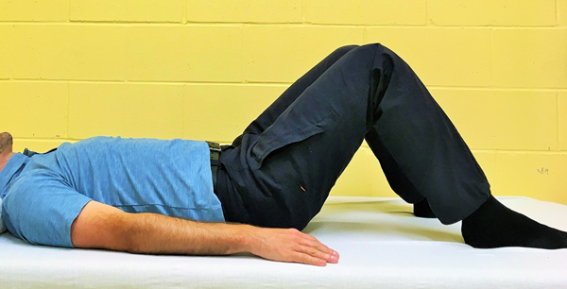Or  **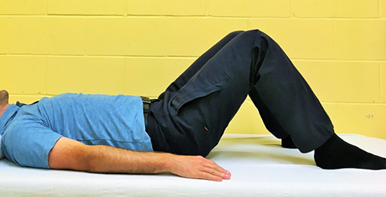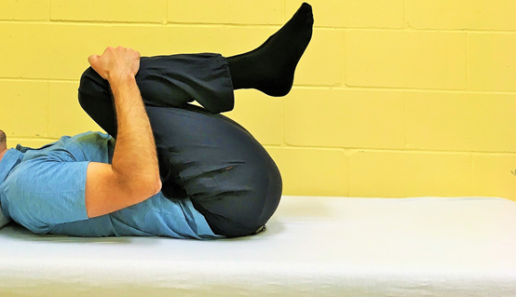** |
| 1. **Child’s pose**  - Start in a 4-point kneeling position. - Bring your pelvic backwards and sit on your heels. - Maintain for 15 to 30 seconds. | 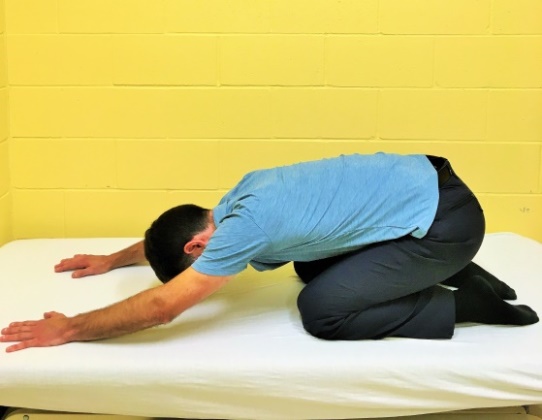  **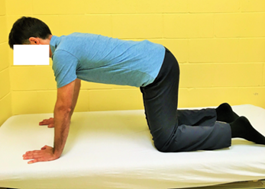** |
| 1. **Spine rotation supine**  - Start lying on your back, with your knees bent. - Bring slowly your knees to one side, hold for 5 to 10 seconds, and come back to the initial position. - Start again on the other side. | 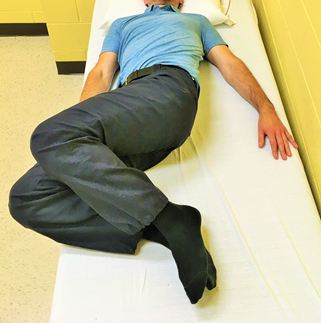  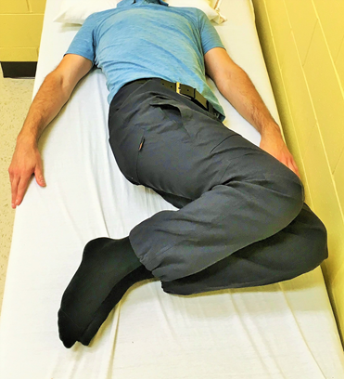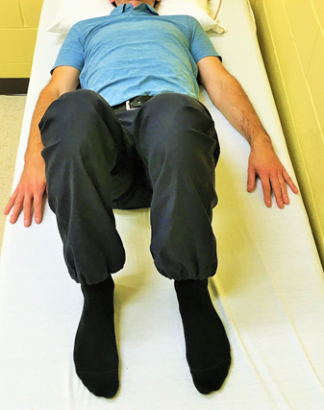 |
| 1. **Spine rotation sitting or standing**  - Start in a sitting position, with your arms crossed on your chest. - Turn your trunk / shoulders towards one side until you feel a gentle stretch in the spine. Hold for 5 seconds and come back to the initial position. - Start again on the other side. - You can also perform this exercise in a standing position. | **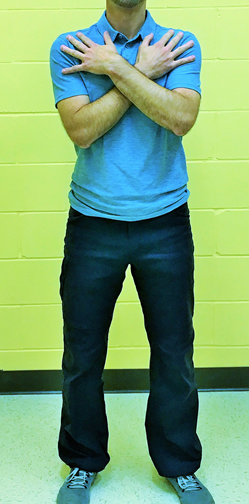**  **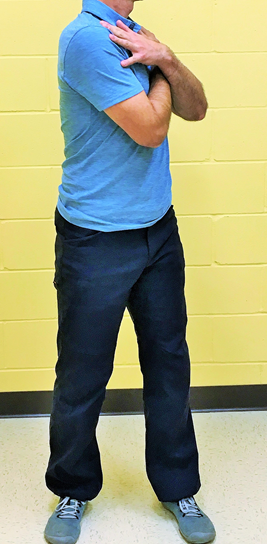**  Or  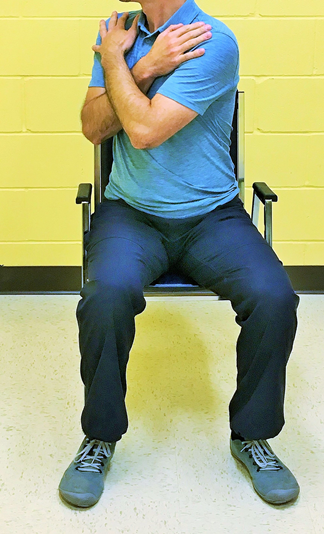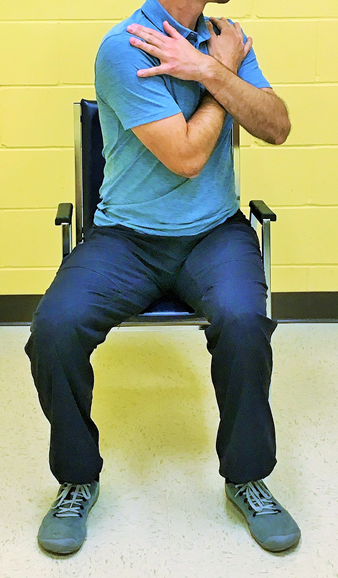**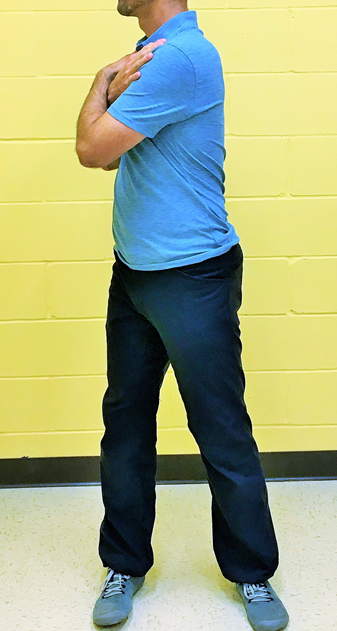**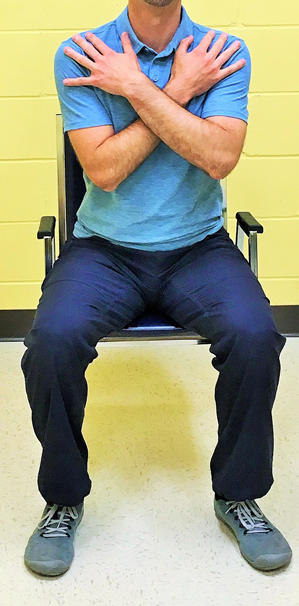 |
| 1. **Side bending**  - Start in a standing position, with your hands on the side of your thighs. - Bend slightly your trunk on one side, by sliding your hand on your thigh. - Hold for 5 seconds and come back to the initial position. - Start again on the other side. | **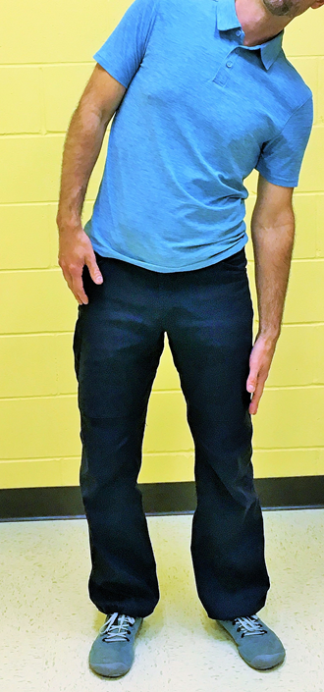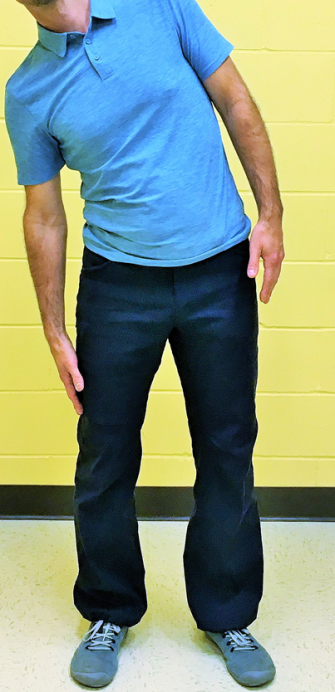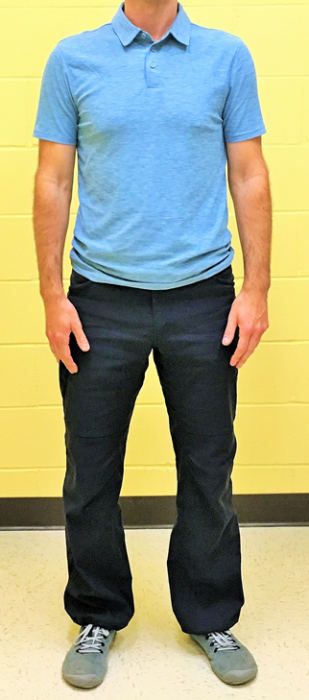** |
| 1. **Shift correction**  - Stand against a wall, on the side that is your shift. - Place the hand of the opposite side on your hip, and push your pelvis towards the wall. - Maintain for 5 to 10 seconds, without increasing the pain, and come back to the initial position. | **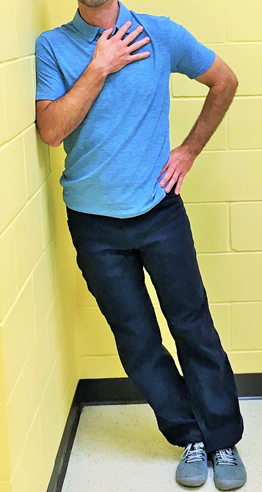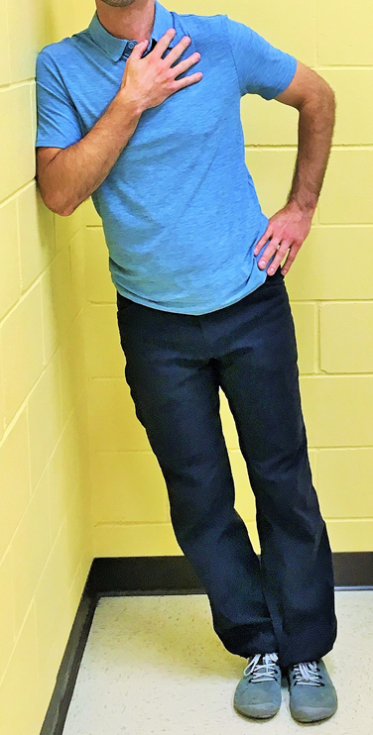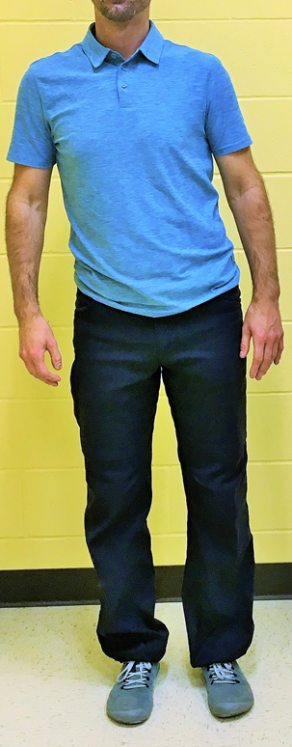** |
| **D- Preferential direction** | |
| 1. **Standing extension**  - Start in a standing position, with your hands placed on your buttock. - Bend your trunk backwards. - Maintain for 5 seconds, and come back to the initial position.   Or   - Variation: perform the same exercise, but with your elbows lying against a wall. | 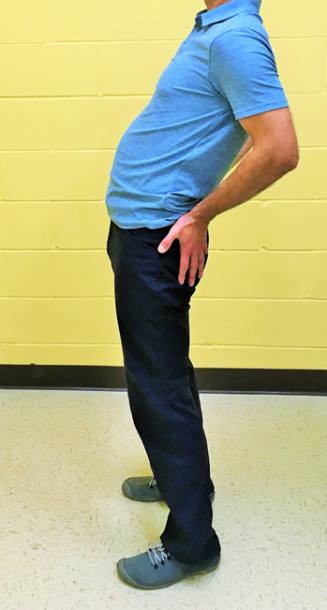**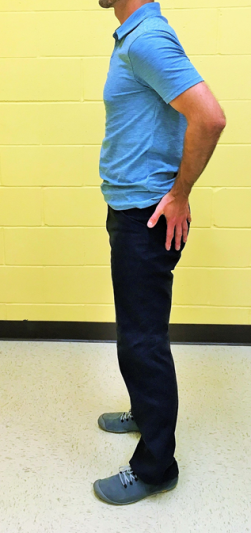**  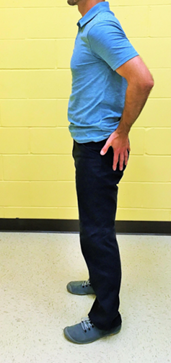Or  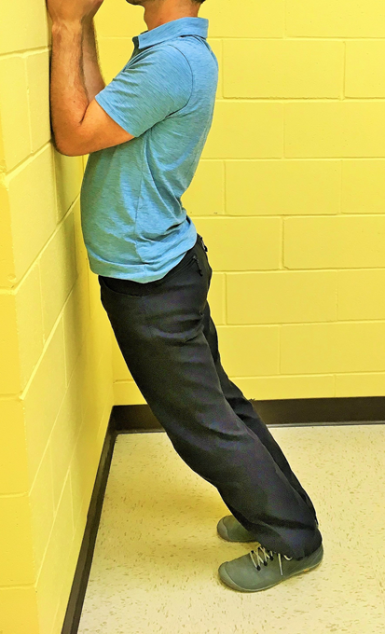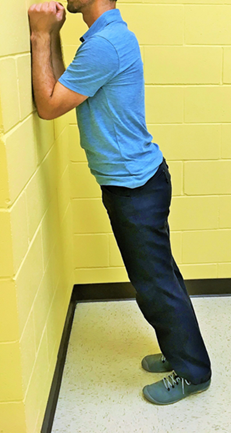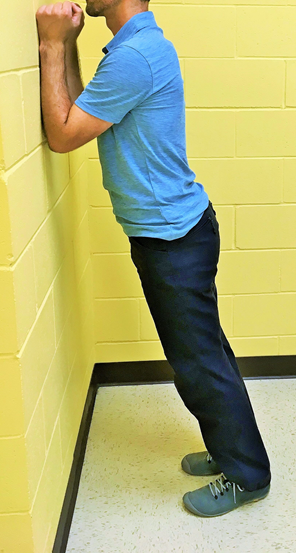 |
| 1. **Flexion**  - Start in a standing position. - Bend your trunk forwards, letting your hands fall. - Maintain for 5 seconds, and come back to the initial position. | **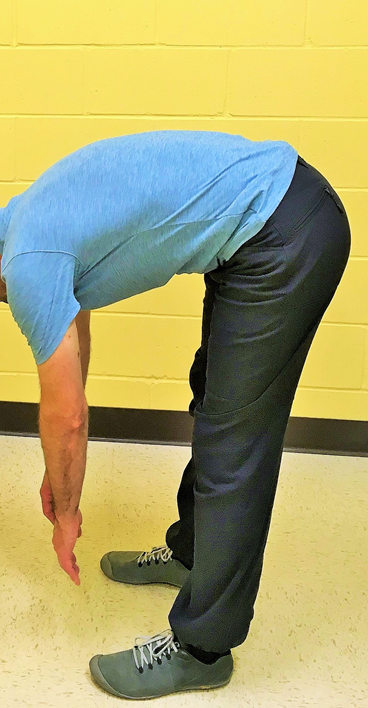**  **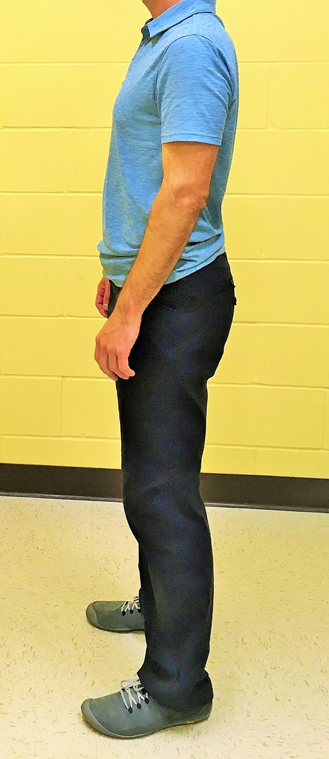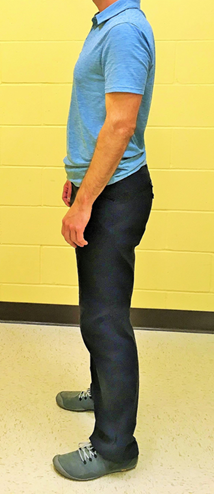** |

| 1. **Puppy / cobra**  - Start lying on your belly. - Lift your upper back up, supported by your elbows or your hands. - Maintain for 5 to 10 seconds, and come back to the initial position. | 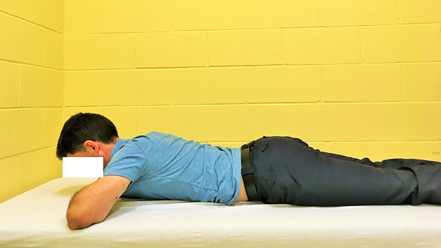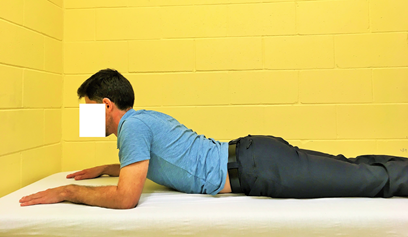Or  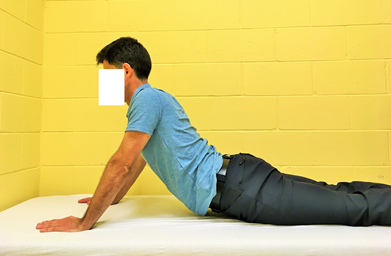  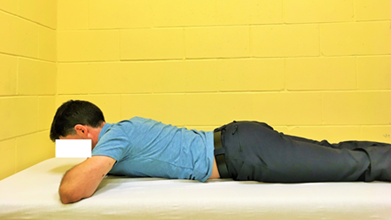 |
| --- | --- |
